# Hematological Ratios in *Leishmania infantum*—Seropositive and Seronegative Dogs and Their Distribution Across Clinical Stages

**DOI:** 10.3390/ani16040568

**Published:** 2026-02-12

**Authors:** Miquel Monroig, Paula F. Navarro, Rebeca Movilla, Blanca Díaz, Maria Dolores Tabar-Rodriguez, Quinidio Melero, Ariadna Ribas, Ignacio Mesa, Jorge Castro-López, Laura Gil-Vicente

**Affiliations:** 1 Doctoral School, Universidad Católica de Valencia San Vicente Mártir, 46001 Valencia, Spain; 2 Department of Animal Medicine and Surgery, Universidad Católica de Valencia San Vicente Mártir, 46001 Valencia, Spain; 3 Internal Medicine Department, Puchol Hospital Veterinario, 28050 Madrid, Spain; 4 Internal Medicine Department, AniCura San Vicente Hospital Veterinario, 03690 Alicante, Spain; 5 Internal Medicine Department, Veterios Hospital Veterinario, 28022 Madrid, Spain; 6 Internal Medicine Department, IVC Evidensia Aúna Especialidades Veterinarias, 46980 Valencia, Spain; 7 Internal Medicine Department, AniCura Valencia Sur Hospital Veterinario, 46460 Valencia, Spain; 8 Internal Medicine Department, Hospital Veterinario UCV, 46018 Valencia, Spain

**Keywords:** biomarkers, canine, hematological inflammatory ratios, *Leishmania infantum*, MLR, NLR, PLR, SII

## Abstract

The neutrophil-to-lymphocyte ratio (NLR), platelet-to-lymphocyte ratio (PLR), monocyte-to-lymphocyte ratio (MLR), and systemic immune-inflammation index (SII) are derived hematological indices reflecting systemic inflammatory balance in dogs with different serological statuses for canine leishmaniosis (CanL). The aim of this study was to describe and compare hematological ratios (HRs) between dogs seropositive and seronegative for *Leishmania infantum* and to assess their distribution across LeishVet stages. A retrospective multicenter case–control study was conducted, including 305 seropositive dogs and 305 seronegative controls from referral hospitals in Spain. Hematological data were retrospectively reviewed and analyzed. Significant differences were observed in NLR and MLR between seronegative control dogs and dogs with leishmaniosis, whereas no differences were found for PLR or SII. Median NLR and MLR values were higher in dogs with leishmaniosis compared to controls. In addition, comparisons across LeishVet stages revealed statistically significant differences for all evaluated HRs. These findings indicate that NLR and MLR are increased in dogs with CanL and that HR varies across LeishVet stages, as these indices represent the relative proportions of neutrophils and monocytes in relation to lymphocytes; therefore, changes in their values reflect shifts in leukocyte distribution observed across clinical stages.

## Simple Summary

Canine leishmaniosis is a widespread parasitic disease in dogs that can involve a wide range of clinical signs, from mild illness to severe, life-threatening conditions. Inflammation plays an important role in the progression of this disease, and simple blood tests may help to evaluate its presence and severity. In this study, we compared routine blood cell ratios that reflect inflammation between dogs with and without canine leishmaniosis, as well as among different stages of the disease. Data from more than 600 dogs from veterinary hospitals across Spain were analyzed. Significant differences were observed between healthy dogs and dogs with canine leishmaniosis in blood cell ratios that compare different types of white blood cells. In particular, ratios comparing neutrophils and monocytes with lymphocytes were higher in dogs with the disease, while ratios involving platelets did not show relevant differences. In addition, these blood cell ratios varied depending on how advanced the disease was. These results suggest that simple and inexpensive blood measurements obtained during routine laboratory testing may provide useful information about inflammation and disease progression in dogs with canine leishmaniosis, potentially helping veterinarians in the clinical evaluation and monitoring of affected animals.

## 1. Introduction

Canine leishmaniosis (CanL) is an important zoonotic disease caused by *Leishmania infantum*, transmitted through the bite of infected phlebotomine sand flies. Dogs are considered the main domestic reservoir, and the disease is endemic in over 70 countries worldwide. Infection with *L. infantum* can result in a chronic and severe disease that may eventually prove fatal in dogs. Clinical manifestations and clinicopathological abnormalities are highly variable, complicating diagnosis, staging, and monitoring of affected dogs [1,2,3,4]. Hematological changes commonly observed in CanL include mild, non-regenerative normocytic anemia associated with chronic inflammation and iron sequestration within macrophages, and more severe anemia in cases with concurrent chronic kidney disease [5,6]. White blood cell and platelet alterations, such as neutrophilia, lymphopenia, lymphocytosis, thrombocytopenia, or thrombocytosis, reflect inflammatory activity and disease progression [2,3,5,6,7,8].

However, individual hematological parameters in CanL are often variable and may not consistently reflect the overall balance between different leukocyte subsets and platelets. In this context, integrated hematological ratios (HRs) that combine several cell lines into a single index may provide a more stable and informative picture of systemic inflammation. Emerging systemic inflammatory indices, hereafter referred to as HR, such as the neutrophil-to-lymphocyte ratio (NLR), monocyte-to-lymphocyte ratio (MLR), platelet-to-lymphocyte ratio (PLR), and systemic inflammation index (SII), have gained attention as accessible biomarkers of inflammation. These ratios have been extensively studied in human medicine and have been associated with prognostic or diagnostic information in diseases such as cancer, cardiovascular disease, vestibular disorders, obstructive pulmonary disease, and COVID-19 [9,10,11,12,13,14,15,16,17,18,19,20,21,22,23]. Different HRs, such as NLR and PLR, have been studied in human patients with leishmaniasis [24,25,26]; they can distinguish healthy individuals from those with leishmaniasis, with both ratios being higher in diseased patients, and they allow assessment of the response to antimonial therapy [24,26]. These HRs have been studied in veterinary medicine across various diseases, including pancreatitis, myxomatous mitral valve disease, chronic enteropathy, parvoviruses, leptospirosis, meningoencephalitis of unknown etiology, and cancer, showing differences between healthy and affected dogs [27,28,29,30,31,32,33,34,35,36,37,38,39]. In addition, reference intervals for several HRs, including NLR, MLR, PLR, and the systemic immune-inflammation index (SII), have recently been established in healthy dogs, providing a baseline framework for their clinical interpretation [40].

In veterinary medicine, four articles have compared different HR values between populations of leishmaniosis-seropositive dogs and leishmaniosis-seronegative dogs, observing higher HR values in seropositive dogs compared to seronegative dogs [41,42,43,44]. Recently, a study found that MLR, NLR, and PLR are elevated in stage II compared to healthy positive dogs, while MLR and NLR increase in stages III/IV compared to negative dogs or healthy positive dogs [41]. These indices represent accessible HR that reflect systemic inflammatory responses and have been explored in various clinical contexts.

Although several studies have evaluated HRs in dogs with CanL, most have relied on relatively small sample sizes, focused on selected clinical stages, or did not systematically compare seropositive and seronegative populations across the full LeishVet spectrum. In addition, the distribution of these ratios across disease stages and their relationship with underlying leukocyte counts have not been consistently assessed in large, multicenter cohorts.

The aim of this study was to describe and compare HRs between dogs seropositive and seronegative for *L. infantum*, as well as to assess their distribution across the different LeishVet stages. The hypothesis was that dogs in the seropositive group would exhibit higher HR values and that those in more advanced LeishVet stages would show elevated levels of these ratios.

## 2. Materials and Methods

This retrospective multicenter case–control study was conducted using data collected between 2018 and 2024 from six referral veterinary hospitals in Spain: AniCura San Vicente Hospital Veterinario (San Vicente del Raspeig, Alicante), AniCura Valencia Sur Hospital Veterinario (Silla, Valencia), Hospital Veterinario UCV (Valencia), IVC Evidensia Aúna Especialidades Veterinarias (Paterna, Valencia), Puchol Hospital Veterinario (Madrid), and Veterios Hospital Veterinario (Madrid).

Dogs were classified into two groups based on the presence or absence of antibodies against *L. infantum*: *L. infantum*–seropositive dogs (LDs) and *L. infantum*–seronegative dogs (CDs). LDs with concurrent conditions, such as neoplasia or systemic infections, or those receiving anti-inflammatory treatment when sampled, were excluded. Dogs in the CD group were recruited from the CEDIVET Iberia laboratory database and originated from annual vector-borne disease screening campaigns. Of the seronegative control dogs, 49% were included in a previous reference interval study of HRs [40], and the new dogs were incorporated from the same laboratory database.

The diagnosis of LDs was based on positive serology for *L. infantum* antibodies using indirect immunofluorescence or ELISA and supported by complete physical examination, complete blood count (CBC), serum biochemistry, protein electrophoresis, and urinalysis, including the urine protein-to-creatinine ratio. In selected cases, cytology or PCR was used to confirm infection. As all dogs in the LD group presented positive serology along with clinical signs or clinicopathological abnormalities consistent with the disease, they were classified according to the LeishVet guidelines. CDs were considered healthy based on normal CBC, serum biochemistry, serology, and protein electrophoresis results. Physical examination data were unavailable due to the retrospective nature of the database.

CBCs were performed using five different hematology analyzers: the ADVIA 2120i CBC analyzer (Siemens Healthcare GmbH, Munich, Germany; Hospital Veterinario UCV), the Celltac Alpha VET MEK-6550 (Nihon Kohden Ibérica, Madrid, Spain; CEDIVET Iberia, Valencia, Spain), the Mindray BC-5000 Vet Hematology Analyzer (Mindray Medical International Limited, Shenzhen, Guangdong, China; Puchol Hospital Veterinario), the ProCyte IDEXX (IDEXX Laboratories, Hoofddorp, the Netherlands; Hospital Veterinario UCV, IVC Evidensia Aúna Especialidades Veterinarias, AniCura Valencia Sur Hospital Veterinario, Veterios Hospital Veterinario, and AniCura San Vicente Hospital Veterinario), and the Sysmex XN-1000 Hematology Analyzer (Sysmex España, Barcelona, Spain; Puchol Hospital Veterinario).

From each CBC, the following parameters were extracted: total leukocyte, neutrophil, monocyte, lymphocyte, and platelet counts. All hematological parameters were expressed in K/µL (thousands of cells per microliter). HRs, including the neutrophil-to-lymphocyte ratio (NLR), monocyte-to-lymphocyte ratio (MLR), platelet-to-lymphocyte ratio (PLR), and systemic immune-inflammation index (SII), were also calculated. NLR, MLR, and PLR were calculated by dividing the total count of the respective cell type by the total lymphocyte count. SII was calculated as the product of the neutrophil-to-lymphocyte ratio and the total platelet count. Blood smear results were excluded because they were not available for all patients.

Data normality was assessed using the Shapiro–Wilk test. As most variables were non-normally distributed, results are presented as medians with the interquartile range, defined as the 25th and 75th percentiles. Statistical significance was set at *p* < 0.05. Differences between LDs and CDs were analyzed using the Mann–Whitney U test. Comparisons across LeishVet stages were performed using the Kruskal–Wallis test, followed by Dunn’s post-hoc test with Bonferroni adjustment; adjusted *p*-values are reported as *p*.adj. Statistical analyses were performed using R software (version 4.4.1; R Development Core Team, Vienna, Austria).

## 3. Results

The LD group consisted of 305 dogs, of which 176 (58%) were male (95 entire and 77 neutered) and 129 (42%) were female (48 entire and 75 spayed). The median age was 7 years (range, 4–9.95 years). Among these dogs, 220 were purebred, representing 60 different breeds, and 85 were mixed-breed. The most represented breeds were American Staffordshire Terrier and Labrador Retriever (19 dogs each), followed by Podenco Andaluz (18 dogs), French Bulldog and American Pit Bull Terrier (10 dogs each), and Dachshund, Belgian Shepherd, and German Shepherd (8 dogs each). According to the LeishVet clinical staging system, 34 dogs were classified as stage I, 170 as stage II, 54 as stage III, and 47 as stage IV.

The CD group consisted of 305 dogs, including 166 males (54%) and 139 females (46%). Information regarding reproductive status, breed, and age was not available for this group.

Significant differences were observed between LDs and CDs in absolute monocyte, lymphocyte, and platelet counts, although all values remained within their respective reference ranges. CDs had higher total lymphocyte and platelet counts, and LDs had higher monocyte counts (Table 1). Nevertheless, the total neutrophil count did not differ significantly between the two populations (Table 1). LDs had significantly higher NLR and MLR than CDs (Table 2). No significant differences were found between the two populations for PLR and SII (Table 2).

The comparison of HR across different LeishVet stages revealed statistically significant differences for all parameters (Table 3). For the NLR, significant differences were observed between LeishVet stages II and III (*p*.adj = 0.02), and II and IV (*p*.adj ≤ 0.001). For the MLR, significant differences were found between stages I and IV (*p*.adj ≤ 0.001), and II and IV (*p*.adj ≤ 0.001). A difference in PLR was noted between stages I and II (*p*.adj = 0.05). For SII, dogs in stage I had higher values than those in stage II (*p*.adj = 0.02), and dogs in stage IV had higher SII values than those in stage II (*p*.adj < 0.001).

## 4. Discussion

This multicenter retrospective study is consistent with previous observations showing that LDs have higher NLR and MLR than CDs, while PLR and SII do not show consistent differences between groups. In addition, the present study expands existing data by describing the distribution of these ratios across LeishVet clinical stages in a large multicenter cohort. Compared with previous studies [41,42,43,44], the main contribution of this work lies in the large sample size, the multicenter design, and the structured comparison across LeishVet stages, which allow a more robust characterization of HR behavior along the clinical spectrum of disease, even though the overall patterns largely reproduce findings reported in smaller cohorts.

Analysis of leukocyte populations in the present study showed that the median total neutrophil count was very similar between CDs and LDs, whereas LDs exhibited higher monocyte counts and lower lymphocyte counts compared with CDs (Table 1). These findings are consistent with previous reports in naturally infected dogs [6]. In canine leishmaniosis, the immune response—regulated by inflammatory cells and parasite-induced chemical mediators—plays a central role in disease development and progression [45,46,47]. The early phases of the immune response are characterized by the recruitment of phagocytic cells to the site of infection, with neutrophils representing the predominant cell type and exhibiting significant leishmanicidal activity [48,49,50]. Neutrophils exert a dual role: they can directly eliminate the parasite but may also inadvertently facilitate its dissemination by acting as “Trojan horses.” In this mechanism, *L. infantum* promastigotes are phagocytosed by neutrophils, which are subsequently engulfed by macrophages, thereby providing a protected environment for parasite survival and replication. In addition, neutrophils can release neutrophil extracellular traps, a form of controlled cell death aimed at containing the infection. These mechanisms may contribute to the maintenance of relatively stable circulating neutrophil counts despite marked immune activation [51,52,53]. By contrast, another study [54] reported higher neutrophil counts in infected dogs compared with healthy dogs, a discrepancy that may be explained by differences in disease stage or by the smaller sample size of that study.

Active CanL induces marked systemic inflammation and stress, leading to alterations in leukocyte populations and elevated inflammatory markers. One of the critical indicators of systemic inflammation is the NLR, which is often elevated in response to infection [43,45,55]. The increase in NLR may stem from neutrophilia, driven by the mobilization of neutrophils to combat the parasite, or lymphopenia, frequently observed in chronic inflammatory conditions [43,45,55]. In the context of CanL, lymphopenia could result from the redistribution of lymphocytes to lymphoid tissues or their apoptosis induced by pro-inflammatory cytokines [3,43]. Thus, even in cases where neutrophil counts remain within normal limits, a significant lymphocyte reduction can lead to an elevated NLR. This underscores the complexity of CanL pathogenesis, with multiple immune mechanisms contributing to hematological changes. Understanding these dynamics is important for the descriptive interpretation of hematological changes observed in CanL [43]. In our population, LDs showed similar median total neutrophil counts but lower lymphocyte counts compared to CDs, suggesting that lymphopenia rather than neutrophilia is the main driver of the increased NLR observed in LDs (Table 1 and Table 2).

The difference in the total monocyte count between the two populations may be because monocytes and macrophages are the primary target cells for the *Leishmania* parasite. As a result, the bone marrow continuously produces monocytes to replace the destroyed macrophages [56,57]. The higher monocyte counts and increased MLR observed in LDs compared to CDs in this study are consistent with enhanced monocyte/macrophage recruitment and turnover in CanL.

Consistent with previous studies assessing HR in *L. infantum*–positive humans and dogs, higher NLR [24,41,42,43] and MLR [41] values have been reported in LD patients compared with controls. Similarly, in the present study, differences in these parameters were also observed between CDs and LDs, as well as across disease stages.

With respect to PLR and SII, both indices incorporate platelet and lymphocyte counts, while SII additionally includes neutrophils. Both PLR and SII are indirect indicators of the organism’s immune-inflammatory response. In the present study, no statistically significant differences were observed between CDs and LDs for these two indices, whereas previous studies have reported differences in SII but not in PLR between these populations [41,42]. The absence of statistically significant differences in these ratios between CDs and LDs in this study may be explained by the generally limited impact of platelet count alterations on the hematological profile of CanL, as platelet abnormalities are typically mild and infrequent [6], with most affected dogs presenting platelet counts within the reference interval. In addition, a potential interference due to platelet aggregates, which could not be systematically excluded in this retrospective dataset, may also have contributed to the lack of significant differences observed.

When considering the different LeishVet clinical stages, NLR values were lower in dogs classified as stage II compared with those in stages III and IV, indicating a progressive increase in this ratio across more advanced clinical stages. Similarly, MLR values were significantly higher in dogs at stage IV than in those at stages I and II, indicating a predominance of monocytes in more advanced stages of CanL. According to the LeishVet classification [2], more advanced stages are associated with a more severe disease. The increase in these two ratios observed in the most severe stages of the classification may have different causes. On the one hand, as the disease progresses in dogs with a TH2 response, the parasite spreads throughout the body, leading to an increase in antibody production. This increase, combined with high parasitemia, results in the formation of immune complexes. These immune complexes are deposited in certain organs, such as the kidneys, joints, skin, and eyes, triggering a more intense inflammatory response [3,5,52,58]. A previous study [59] has demonstrated that as the disease progresses, the number of circulating immune complexes increases, which is directly related to the patient’s worsening condition. On the other hand, in the chronic stages of the disease, T lymphocyte exhaustion may occur, resulting in lymphopenia [3,6,43,53]. Consistent with these mechanisms, we found higher NLR and MLR values in dogs classified in LeishVet stages III and IV compared with those in stages I and II, indicating that these ratios reflect shifts in leukocyte distribution observed across advanced clinical stages (Table 3). In one study [41], it was observed that patients with clinical disease, including those at stages II and III/IV, had higher concentrations of NLR and MLR compared to healthy-positive patients. These results are similar to those obtained in the present study, indicating that these parameters vary across different stages of the LeishVet classification.

With respect to PLR, it has been observed that patients in stage I present a higher ratio compared to patients in stage II. This association could be explained by the fact that patients in stage I do not show evident clinicopathological alterations, whereas from stage II onward, changes in the CBC begin to be observed. As a result, the counts of platelets and lymphocytes could be different in stage I compared to the other stages of the LeishVet classification [2]. In contrast to what was reported previously [41], this study did not observe a significant difference between sick patients or those in higher LeishVet stages and those in lower stages. Therefore, further research is needed to evaluate the impact of PLR at different stages of LeishVet.

Finally, patients in stages I and IV present higher concentrations of SII compared to those in stage II. Since this ratio depends on the concentrations of neutrophils, platelets, and lymphocytes, it is difficult to determine which of these factors contributes to the higher ratio values in stages I and IV compared to stage II. Therefore, further studies specifically designed to control for leukocyte and platelet variability are needed to clarify these findings.

The main limitation of this study is its retrospective and descriptive design, which is inherently associated with potential sources of bias, including selection bias, information bias, and residual confounding. In particular, dogs in the seropositive and control groups were not matched by age or breed, and detailed demographic and clinical information—such as age, sex, reproductive status, clinical examination findings, and comorbidities—was unavailable for the seronegative control group. As these factors can influence leukocyte and platelet counts and derived HR, it cannot be determined to what extent the observed differences between groups are attributable solely to infection status rather than to underlying population differences. For this reason, multivariable analyses were not performed, as the inclusion of incomplete or unevenly distributed covariates would have resulted in biased or unreliable models. Accordingly, all comparisons should be interpreted with caution, and no assumption of demographic equivalence between groups should be made.

In addition, hematological data were generated using five different automated analyzers across participating centers. Although all instruments employed flow cytometry-based methods for leukocyte differentiation, inter-analyzer variability may have affected absolute cell counts and, consequently, the calculated ratios. No formal harmonization or cross-calibration of counts between analyzers was performed in this retrospective dataset, which further limits the precision of between-group and between-stage comparisons. Furthermore, blood smear evaluation was not available for all patients and was therefore excluded from the analysis. This limitation prevented confirmation of automated differential counts and assessment of platelet aggregates, which may have affected platelet-derived ratios, particularly PLR and SII.

## 5. Conclusions

In conclusion, NLR and MLR were higher in LDs compared with CDs, reflecting a tendency toward lower lymphocyte counts and higher monocyte counts in LDs. In addition, variations were observed in all evaluated HRs across LeishVet clinical stages, with higher NLR and MLR values in more advanced stages, consistent with progressive inflammatory and immunopathological changes observed across LeishVet clinical stages. These findings should be interpreted as descriptive observations. Further prospective studies controlling for potential confounding factors are needed to clarify the clinical relevance of these HRs in canine leishmaniosis; nevertheless, NLR and MLR may represent accessible, low-cost markers that could complement existing tools for the clinical assessment and monitoring of dogs with leishmaniosis.

## Figures and Tables

**Table 1:** Description of total leukocyte counts in *Leishmania infantum*-seropositive dogs (LDs) versus *Leishmania infantum*-seronegative dogs (CDs).

Parameter	Reference Range (K/μL)	CD Median (25th and 75th Percentiles)	LD Median (25th and 75th Percentiles)	*p*-Value
Total neutrophil count	3.0–12.0	6.80 (5.50–8.10)	6.61 (4.70–9.60)	0.68
Total monocyte count	<2.0	0.30 (0.20–0.40)	0.60 (0.39–1.00)	<0.001
Total lymphocyte count	0.5–4.9	2.30 (1.70–2.90)	1.69 (1.17–2.36)	<0.001
Total platelet count	175–500	242 (196–312)	210 (134.80–277.20)	<0.001

K/μL = thousands of cells per microliter.

**Table 2:** Statistical comparison of hematological ratios in *Leishmania infantum*-seropositive dogs (LDs) versus *Leishmania infantum*-seronegative dogs (CDs).

Parameter	CD Median (25th and 75th Percentiles)	LD Median (25th and 75th Percentiles)	*p*-Value
NLR	3 (2.25–3.93)	3.95 (2.44–6.56)	<0.001
MLR	0.14 (0.12–0.19)	0.37 (0.22–0.61)	<0.001
PLR	113.45 (78.48–147.27)	114.40 (68.15–173.51)	0.67
SII	710.91 (512.31–1085.14)	758.10 (406.30–1364.90)	0.77

**Table 3:** Statistical description of HR in dogs at different LeishVet stages.

Parameter	LeishVet I Median (25th and 75th Percentiles)	LeishVet II Median (25th and 75th Percentiles)	LeishVet III Median (25th and 75th Percentiles)	LeishVet IV Median (25th and 75th Percentiles)	*p*.adj
NLR	4.20 (2.65–6.12)	3.35 (2.09–5.16)	4.62 (2.73–7.29)	6.72 (4.09–9.45)	<0.001
MLR	0.33 (0.24–0.45)	0.31 (0.20–0.53)	0.40 (0.24–0.73)	0.63 (0.40–0.84)	<0.001
PLR	145 (84–214)	105 (55–159)	116 (97–185)	134 (76–205)	<0.001
SII	988 (670–1492)	600 (320–1170)	947 (452–1732)	1082 (640–1955)	<0.001

*p*.adj = Bonferroni-adjusted *p*-value from Dunn’s post-hoc test.

## Data Availability

The original contributions presented in this study are included in the article. Further inquiries can be directed to the corresponding authors.

## Abbreviations

The following abbreviations are used in this manuscript:

CanL	Canine leishmaniosis
CBC	Complete blood count
CD	*Leishmania infantum*-seronegative dog
HR	Hematological ratio
LD	*Leishmania infantum*-seropositive dog
*L. infantum*	*Leishmania infantum*
MLR	Monocyte-to-lymphocyte ratio
NLR	Neutrophil-to-lymphocyte ratio
PLR	Platelet-to-lymphocyte ratio
SII	Systemic immunity–inflammation index
